# Supplementary material for: Hot spot prediction in protein-protein interactions by an ensemble system
Source: BMC Syst Biol. 2018 Dec 31;12(Suppl 9):132. doi: 10.1186/s12918-018-0665-8 (PMC6311905; doi:10.1186/s12918-018-0665-8)
Supplement: Supplementary file 2 — All relevant MATLAB code. A simple Matlab implement of our predictor. (ZIP 19,742 kb) [file 12918_2018_665_MOESM2_ESM.zip › Additional file 2/Read ASA/ASA Property/SKEMPI/1LFDA.txt]

 NetSurfP - Protein Surface Accessibility and Secondary Structure Predictions

  

|  |  |  |
| --- | --- | --- |
|  |  | NetSurfP - Protein Surface Accessibility and Secondary Structure Predictions  Technical University of Denmark |

  


---

```
# For publication of results, please cite:
# A generic method for assignment of reliability scores applied to solvent accessibility predictions.
# Bent Petersen, Thomas Nordahl Petersen, Pernille Andersen, Morten Nielsen and Claus Lundegaard
# BMC Structural Biology 2009, 9:51 doi:10.1186/1472-6807-9-51
#
# Column 1: Class assignment - B for buried or E for Exposed - Threshold: 25% exposure, but not based on RSA
# Column 2: Amino acid
# Column 3: Sequence name
# Column 4: Amino acid number
# Column 5: Relative Surface Accessibility - RSA
# Column 6: Absolute Surface Accessibility
# Column 7: Z-fit score for RSA prediction
# Column 8: Probability for Alpha-Helix
# Column 9: Probability for Beta-strand
# Column 10: Probability for Coil
E G  1LFDA                  1    0.783  61.638  -0.405   0.003   0.003   0.994
E D  1LFDA                  2    0.438  63.145  -0.609   0.004   0.138   0.858
B C  1LFDA                  3    0.069   9.716  -0.629   0.004   0.616   0.381
B C  1LFDA                  4    0.100  14.012   0.598   0.001   0.900   0.099
B I  1LFDA                  5    0.043   8.029  -0.090   0.001   0.959   0.040
B I  1LFDA                  6    0.029   5.402   0.425   0.000   0.983   0.017
B R  1LFDA                  7    0.117  26.862   0.602   0.000   0.983   0.017
B V  1LFDA                  8    0.029   4.442   0.479   0.001   0.959   0.040
B S  1LFDA                  9    0.101  11.896  -0.102   0.001   0.959   0.040
B L  1LFDA                 10    0.063  11.535   0.634   0.001   0.900   0.099
E D  1LFDA                 11    0.322  46.415   0.319   0.002   0.816   0.182
B V  1LFDA                 12    0.246  37.779  -0.438   0.005   0.262   0.733
E D  1LFDA                 13    0.656  94.544  -1.200   0.004   0.085   0.910
E N  1LFDA                 14    0.613  89.787  -0.440   0.005   0.015   0.979
B G  1LFDA                 15    0.213  16.747  -0.991   0.018   0.088   0.893
E N  1LFDA                 16    0.290  42.383  -0.178   0.021   0.451   0.528
B M  1LFDA                 17    0.104  20.750   0.664   0.018   0.846   0.136
B Y  1LFDA                 18    0.088  18.848   0.692   0.011   0.918   0.071
B K  1LFDA                 19    0.159  32.747   0.318   0.018   0.846   0.136
B S  1LFDA                 20    0.037   4.313  -0.816   0.011   0.918   0.071
B I  1LFDA                 21    0.031   5.735   0.086   0.011   0.918   0.071
B L  1LFDA                 22    0.111  20.379   0.479   0.011   0.918   0.071
B V  1LFDA                 23    0.041   6.256   0.090   0.021   0.756   0.223
B T  1LFDA                 24    0.192  26.561  -0.396   0.022   0.552   0.426
E S  1LFDA                 25    0.284  33.297  -1.088   0.020   0.205   0.775
E Q  1LFDA                 26    0.468  83.496  -0.926   0.018   0.047   0.935
E D  1LFDA                 27    0.472  68.030  -0.963   0.058   0.017   0.925
E K  1LFDA                 28    0.353  72.633  -0.456   0.181   0.016   0.803
B A  1LFDA                 29    0.163  17.919   0.071   0.257   0.016   0.727
B P  1LFDA                 30    0.138  19.582  -0.240   0.923   0.002   0.076
E T  1LFDA                 31    0.396  54.911   0.577   0.970   0.001   0.030
B V  1LFDA                 32    0.074  11.435   0.230   0.970   0.001   0.030
B I  1LFDA                 33    0.030   5.642   0.458   0.970   0.001   0.030
E R  1LFDA                 34    0.285  65.288   0.999   0.970   0.001   0.030
E K  1LFDA                 35    0.364  74.793   1.295   0.970   0.001   0.030
B A  1LFDA                 36    0.042   4.672  -0.198   0.970   0.001   0.030
B M  1LFDA                 37    0.074  14.807  -0.212   0.970   0.001   0.030
E D  1LFDA                 38    0.452  65.104   0.221   0.970   0.001   0.030
E K  1LFDA                 39    0.466  95.856   0.411   0.858   0.002   0.139
B H  1LFDA                 40    0.214  38.945   0.219   0.600   0.003   0.397
E N  1LFDA                 41    0.493  72.234  -1.337   0.109   0.005   0.886
B L  1LFDA                 42    0.168  30.688  -0.280   0.053   0.005   0.942
E D  1LFDA                 43    0.612  88.132  -0.808   0.016   0.005   0.979
E E  1LFDA                 44    0.645 112.664  -1.233   0.018   0.019   0.964
E D  1LFDA                 45    0.679  97.858  -1.373   0.018   0.019   0.964
E E  1LFDA                 46    0.531  92.801   0.223   0.058   0.017   0.925
E P  1LFDA                 47    0.298  42.286  -0.713   0.058   0.017   0.925
E E  1LFDA                 48    0.603 105.257  -0.233   0.058   0.017   0.925
E D  1LFDA                 49    0.495  71.330   1.130   0.113   0.087   0.800
B Y  1LFDA                 50    0.084  18.036   0.992   0.074   0.484   0.442
B E  1LFDA                 51    0.259  45.177   1.054   0.085   0.775   0.140
B L  1LFDA                 52    0.055  10.089   0.752   0.085   0.775   0.140
B L  1LFDA                 53    0.075  13.678   0.353   0.085   0.775   0.140
B Q  1LFDA                 54    0.142  25.415   0.392   0.085   0.775   0.140
B I  1LFDA                 55    0.124  22.866   0.235   0.023   0.655   0.322
B I  1LFDA                 56    0.065  12.025   1.096   0.022   0.359   0.619
E S  1LFDA                 57    0.352  41.219   0.060   0.019   0.141   0.840
E E  1LFDA                 58    0.667 116.472  -0.331   0.005   0.015   0.979
E D  1LFDA                 59    0.704 101.403  -0.912   0.005   0.015   0.979
E H  1LFDA                 60    0.290  52.787   0.575   0.004   0.138   0.858
E K  1LFDA                 61    0.386  79.441   0.274   0.004   0.616   0.381
B L  1LFDA                 62    0.076  13.916   0.338   0.002   0.816   0.182
E K  1LFDA                 63    0.261  53.626   0.790   0.021   0.756   0.223
B I  1LFDA                 64    0.060  11.026   0.770   0.004   0.420   0.576
B P  1LFDA                 65    0.139  19.767   0.252   0.004   0.085   0.910
E E  1LFDA                 66    0.709 123.775  -0.423   0.005   0.015   0.979
E N  1LFDA                 67    0.531  77.738  -0.625   0.018   0.019   0.964
B A  1LFDA                 68    0.090   9.874  -0.530   0.018   0.019   0.964
B N  1LFDA                 69    0.215  31.491  -0.346   0.019   0.141   0.840
B V  1LFDA                 70    0.067  10.359   0.425   0.023   0.655   0.322
B F  1LFDA                 71    0.054  10.778   0.375   0.018   0.846   0.136
B Y  1LFDA                 72    0.068  14.446   0.335   0.011   0.918   0.071
B A  1LFDA                 73    0.029   3.185  -1.027   0.018   0.846   0.136
B M  1LFDA                 74    0.044   8.744  -0.099   0.021   0.756   0.223
B N  1LFDA                 75    0.095  13.864  -0.060   0.022   0.359   0.619
B S  1LFDA                 76    0.155  18.154  -1.499   0.019   0.141   0.840
B A  1LFDA                 77    0.215  23.660  -1.123   0.052   0.084   0.864
B A  1LFDA                 78    0.261  28.784  -0.443   0.052   0.084   0.864
E N  1LFDA                 79    0.320  46.877  -1.348   0.056   0.142   0.802
B Y  1LFDA                 80    0.152  32.461   0.414   0.064   0.216   0.721
B D  1LFDA                 81    0.244  35.175  -1.268   0.066   0.296   0.638
B F  1LFDA                 82    0.102  20.512   0.404   0.079   0.592   0.329
B I  1LFDA                 83    0.123  22.737  -0.659   0.087   0.683   0.230
B L  1LFDA                 84    0.096  17.578  -0.346   0.087   0.683   0.230
E K  1LFDA                 85    0.332  68.313   0.637   0.021   0.451   0.528
E K  1LFDA                 86    0.499 102.747   0.509   0.020   0.205   0.775
E R  1LFDA                 87    0.795 182.147   0.420   0.003   0.003   0.994

Explain the output.  Go back.
```

---
